# Selected Microelements and Toxic Metals in the Hair of Dairy Cows from the Bishkek Area, Kyrgyzstan

**DOI:** 10.3390/ani16213443

**Published:** 2026-10-31

**Authors:** Slawomir Gonkowski, Paweł Świsłowski, Nariste Kadyralieva, Liliana Rytel, Małgorzata Rajfur

**Affiliations:** 1 Department of Clinical Physiology, Faculty of Veterinary Medicine, University of Warmia and Mazury in Olsztyn, Oczapowskiego 13, 10-957 Olsztyn, Poland; 2 Institute of Biology, University of Opole, Kominka 6a, 45-032 Opole, Poland; pawel.swislowski@uni.opole.pl (P.Ś.); rajfur@uni.opole.pl (M.R.); 3 Department of Histology and Embryology, Veterinary Faculty, Kyrgyz-Turkish Manas University, Bishkek 720044, Kyrgyzstan; nariste.kadyralieva@manas.edu.kg; 4 Department of Internal Diseases with Clinics, Faculty of Veterinary Medicine, University of Warmia and Mazury in Olsztyn, Oczapowskiego 14, 10-957 Olsztyn, Poland; liliana.rytel@uwm.edu.pl

**Keywords:** farm animals, biomonitoring, environmental pollutants, Central Asia

## Abstract

In Kyrgyzstan, despite heavy environmental pollution, no assessment of dairy cow exposure to trace elements using hair sample analysis has been conducted so far. Therefore, this study aimed to assess the concentrations of selected trace elements, including the essential elements Cu, Fe, Mn, and Zn, as well as Cd, Pb, Hg, and Ni, in the hair of dairy cows in the vicinity of Bishkek—the largest city in Kyrgyzstan. The mean concentrations (±SD) and medians (given in parentheses) for individual elements were as follows (in mg/kg dry weight): Cd: 0.62 ± 0.19 (0.60), Cu: 5.63 ± 1.16 (5.69), Fe: 274.9 ± 169.8 (248.0), Mn: 7.81 ± 6.06 (6.12), Ni: mean and median below the limit of detection, Pb: 3.98 ± 1.47 (3.68), Hg: 0.0051 ± 0.0022 (0.0049), and Zn: 77.94 ± 12.91 (79.65). Statistically significant spatial differences were found for Cd, Fe, Hg, Mn, Pb, and Zn, whereas Cu concentrations did not differ significantly among districts. A statistically significant positive correlation with age was found only for Fe (Rs = 0.39, *p* = 0.006). The results demonstrate the occurrence and spatial variability of the analyzed elements in the hair of cows from the vicinity of Bishkek. However, further comprehensive studies are needed to determine the sources of contamination and the influence of the element concentrations noted in this study on animal health status, as well as to assess potential risks to human consumers of animal products.

## Simple Summary

Toxic elements commonly contaminate the natural environment and have an adverse effect on human and animal health. Therefore, monitoring exposure to these elements is a critical aspect of toxicology. Among the matrices used to assess exposure levels, hair samples play an important role because they can integrate toxic substance accumulation over the period represented by hair growth. This study analyzes, for the first time, the levels of selected elements, such as cadmium (Cd), copper (Cu), iron (Fe), manganese (Mn), nickel (Ni), lead (Pb), mercury (Hg), and zinc (Zn) in the hair of dairy cows raised in Kyrgyzstan, specifically in the vicinity of Bishkek—a large industrial center characterized by a high degree of air pollution. The obtained results showed the presence of most of the tested elements in the hair samples collected from the cows included in this study. These findings demonstrate the occurrence of the analyzed elements in cow hair but do not allow their specific sources to be identified. However, the assessment of their potential effects on animal health and possible risks to humans requires further comprehensive research.

## 1. Introduction

Bishkek serves as the capital and the largest metropolitan area of Kyrgyzstan, with a population exceeding one million residents. Consequent to the intensive development of diverse industrial sectors, the city and its periphery exhibit a high degree of environmental degradation [1]. Regarding air pollution in Bishkek, the situation is critical, particularly in winter, when the city is regularly ranked among the top five most polluted cities globally [2]. One of the most important factors contributing to environmental pollution in Bishkek and its surroundings is toxic metals [2,3]. This is due to the intensive development of the metallurgical, metalworking, and mechanical engineering industries. The development of mining also contributes to the high contamination of the environment with metals and other toxic substances [3].

**Figure 1:**
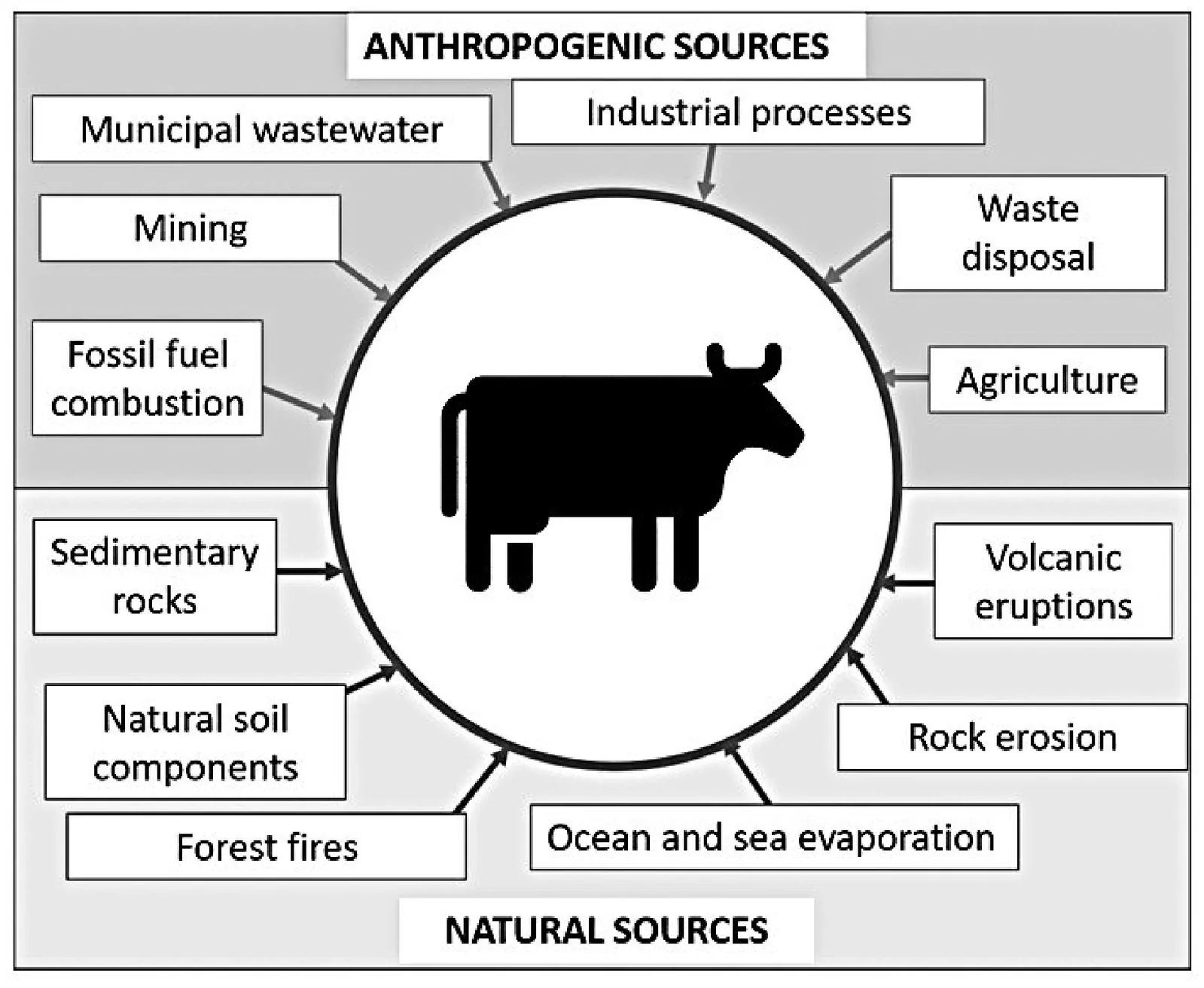
Sources of metal exposure in cows and other living organisms (Source: Author’s own elaboration).

Toxic metals are currently considered to be among the most significant and harmful environmental pollutants both globally and in Bishkek. Although they can enter the environment through natural processes such as erosion, weathering of rocks, or volcanic eruptions, the main source of pollution is human activity related to urbanization, industrialization, and agriculture [4,5] (Figure 1).

Although data regarding toxic metals in the natural environment of Bishkek, as well as the whole of Kyrgyzstan, are relatively limited, it is known that elevated levels of these elements are present both in various parts of the Kyrgyz environment and food, including, among others, Hg, Pb, Cr, and Zn [3,6,7,8,9].

The high content of harmful metals in Kyrgyzstan’s environment is associated with high exposure of humans and animals to these substances. Metals can enter the body mainly through the gastrointestinal tract, but also through the respiratory tract and skin, as well as during fetal life through the placenta [10,11]. It is widely known that exposure to metals can result in the dysfunction of many internal systems and organs, and their toxicity depends on the type of metal, the dose, and the duration of exposure (acute or chronic poisoning), the degree of metal accumulation in the body, as well as the health status and age of the exposed organism [11].

The general mechanism of metal toxicity is based on chemical and structural interactions at the cellular and molecular levels. After entering the living organism, toxic metals readily bind to cellular components, leading to structural damage, metabolic blocks, and systemic functional disorders [12,13]. The pathways of this toxicity include the induction of oxidative stress, interaction with protein sulfhydryl groups resulting in enzyme inhibition, molecular mimicry, and the displacement of essential elements, as well as DNA damage and genotoxicity [12,13,14]. Moreover, it should be underlined that even metals that are essential for the proper functioning of the organism and serve as components of coenzymes can exhibit toxic effects when present in the body in excess [15]. For this reason, a clear classification of metals into essential and harmful is extremely difficult, as the impact of these elements on human and animal health depends primarily on the dose.

The vast majority of previous studies on exposure levels to environmental metal pollutants conducted in Kyrgyzstan concern humans, in whom high levels of these substances have been confirmed in blood serum, hair, and urine [16,17,18]. In contrast, information regarding the exposure of domestic animals to metals in Kyrgyzstan is more limited [19,20], even though this issue is of great importance in contemporary toxicology. Specifically, domestic animals living in close proximity to humans are largely exposed to the same anthropogenic environmental pollutants as humans and therefore can serve as good indicator species, helping to determine the level of such contamination [21,22]. Furthermore, the exposure level of herbivores accurately reflects the levels of harmful metals in plants, as well as in soil and water, as demonstrated by studies conducted in various regions of the world [21,23]. It is also worth noting that determining the level of toxic metals in the bodies of livestock not only aids in analyzing the impact of these elements on animal health and livestock production but also allows for the assessment of potential risks to humans as consumers of animal products [24,25].

Among the matrices in which metal levels are investigated, hair samples deserve special attention. This stems from the fact that hair can provide an integrated measure of exposure to environmental pollutants over the period represented by hair growth. Specifically, it is known that toxic substances (including metals) can deposit in hair, and their levels do not undergo short-term fluctuations, as is the case with other matrices popular in toxicological studies, such as blood serum or urine [26,27]. The incorporation and accumulation of metals in hair result from the fact that hair keratin contains cysteine, whose sulfhydryl (-SH) groups have strong chelating properties, forming stable complexes with metal ions [28,29]. Therefore, hair is a useful matrix for assessing integrated exposure to metals and other anthropogenic environmental pollutants over the hair-growth period [26,27]. Other advantages of hair as a matrix in toxicological studies include the fact that samples can be collected easily, painlessly, and without stress, stored easily even for a long time, and transported over long distances without the need for freezing.

The utility of hair samples for assessing integrated exposure of animals to environmental metal pollutants has been confirmed by previous studies from various regions of the world [22,30,31]. However, it should be noted that despite these undoubted advantages of hair samples, no analysis of metal levels in the hair of livestock in Kyrgyzstan has been conducted to date.

Taking the above into consideration, the objective of this study was to determine, for the first time, the levels of selected metals, including cadmium (Cd), copper (Cu), iron (Fe), manganese (Mn), nickel (Ni), lead (Pb), mercury (Hg), and zinc (Zn) in the hair of dairy cows raised in the vicinity of Bishkek, Kyrgyzstan. The obtained results will complement the existing knowledge regarding the exposure of locally raised dairy cows to these elements and their spatial variability in the Bishkek area, which may be relevant to animal health and potential risks to consumers of animal products.

## 2. Materials and Methods

### 2.1. Sampling

**Figure 2:**
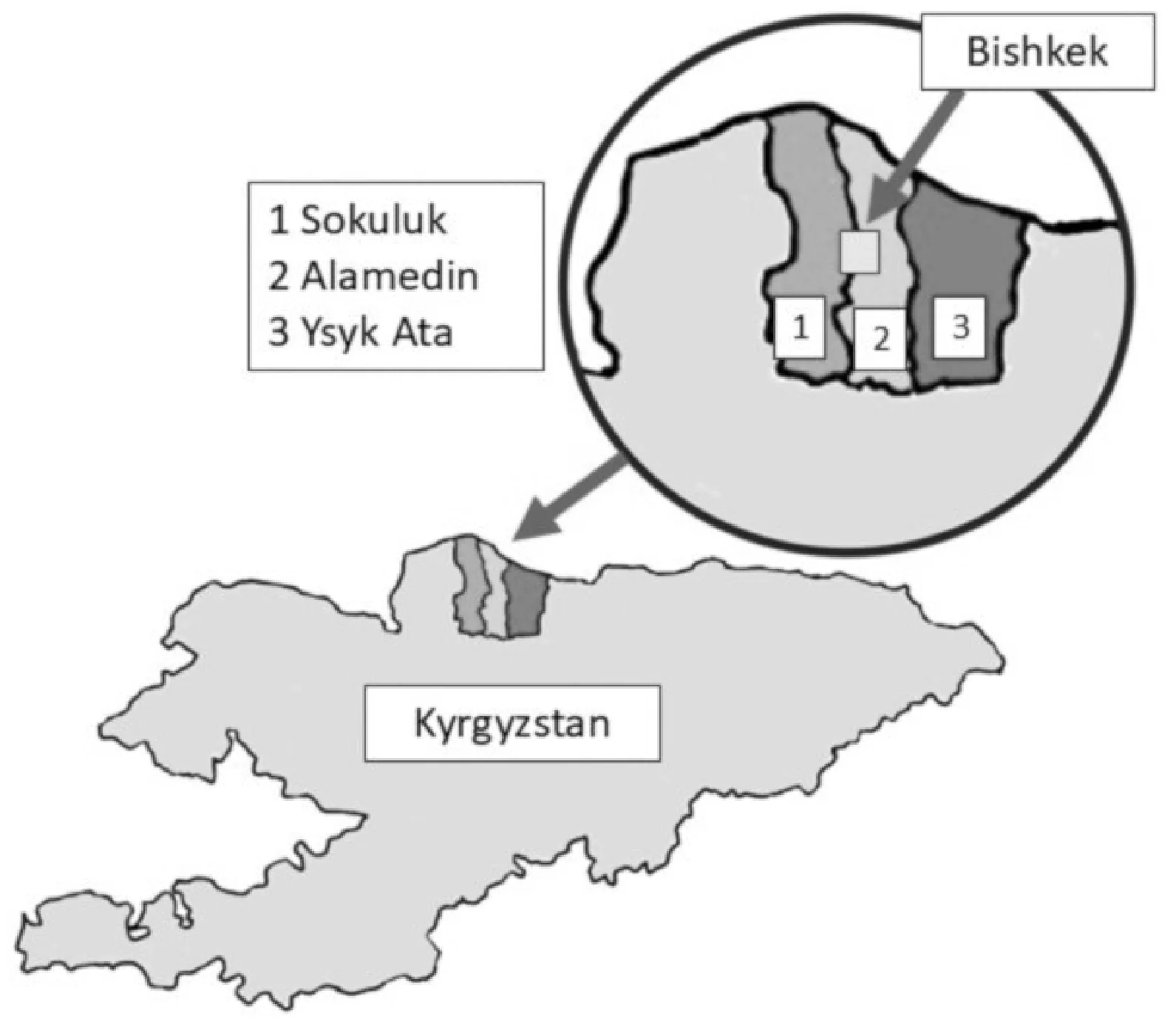
Localization of districts included in this study. Bishkek is located at coordinates 42°52′29″ N 74°36′44″ E (Source: Author’s own elaboration).

This research involved 48 mature dairy cows aged 3 to 8 years, representing various breeds (Alatau, Kyrgyz, and Swiss) and sourced from farms across three districts of the Kyrgyz Republic surrounding the capital and largest urban center, Bishkek, namely Alamedin, Sokuluk, and Ysyk-Ata. Region-specific profiles are summarized in Table 1, and their locations are shown in Figure 2.

Data according to the National Statistical Committee of the Kyrgyz Republic (https://stat.gov.kg, accessed on 20 September 2026) and the Analytical Report on the Inventory of Consumer Waste Disposal Sites within the Territory of the Kyrgyz Republic (in Russian) from 2018 (https://eco-expertise.org/wp-content/uploads/2022/03/Analytical_report_on_the_inventory_of_waste_disposal_sites_.pdf, accessed on 20 September 2026).

The hair was sampled in late May 2023. Given the highly seasonal nature of pastoralism in Kyrgyzstan, where calving typically peaks in early spring (March–April), all examined cows were in their early to peak lactation stage, approximately 30 to 60 days in milk. All cows included in the study were fed a similar diet. From April onwards, they were kept on pastures. Prior to that, in winter and early spring, they were fed mainly hay and concentrate feeds containing barley and corn. The cattle breeds included in the study (namely Alatau, Kyrgyz, and Swiss) are genetically and morphologically similar. Furthermore, the Alatau breed was developed by crossing the Kyrgyz and Swiss breeds. The coat coloration of the cows ranged from dark brown (brown-grey) to light brown. The milk yield of individual cows included in the study was not precisely determined; however, the average milk yield of these cattle breeds in Kyrgyzstan ranges from 8 to 15 L per day. The characteristics of the animals included in the study are presented in the Supplementary Materials, Table S1.

Approximately 2 g of hair with a length of approximately 2.5–3 cm was cut directly at the skin level from a standardized anatomical location (the dorsal region above the scapulae) of each animal. Following collection, the specimens were immediately enclosed in aluminum foil and kept in darkness at ambient temperature pending analysis. Since the sampling process was non-invasive, pain-free, and integrated into routine husbandry practices, ethical committee approval was not legally mandated under Kyrgyz Republic or European Union regulations. The owners of the animals were informed about the aims of the study and gave verbal consent for sampling.

### 2.2. Methodology and Quality Control

Before analysis, the hair samples were cleaned to remove loosely adhering surface contaminants. Each sample was rinsed with 200 mL of deionized water (conductivity: 0.5 µS/cm). No organic solvents were used for defatting. The material was then dried at 38 °C for 48 h to a constant mass. The dried hair was manually cut into small pieces and thoroughly mixed to obtain a representative subsample for analysis. All final concentrations were expressed on a dry-weight basis (dw).

Hair portions of 0.800 ± 0.001 g were digested with 10 mL of 65% HNO_3_ and 6 mL of 30% H_2_O_2_ using a Speedwave Four microwave digestion system (Berghof, Eningen unter Achalm, Germany). A three-stage program was applied. In stage 1, the samples were heated to 145 °C over 10 min and held at this temperature for 5 min at 90% microwave power, with a pressure limit of 40 bar. In stage 2, the temperature was increased to 190 °C over 5 min and maintained for 10 min at 90% microwave power, with a pressure limit of 40 bar. Stage 3 consisted of cooling to 50 °C over 20 min with no microwave power applied. After digestion and cooling, the digests were filtered, quantitatively transferred to 20 mL volumetric flasks, and made up to volume with deionized water.

The concentrations of Mn, Fe, Ni, Cu, Zn, Cd, and Pb were determined by flame atomic absorption spectrometry (F-AAS) using an iCE 3500 atomic absorption spectrometer (iCE 3000 Series; Thermo Scientific, Waltham, MA, USA). One independent microwave digestion was performed for each individual hair sample. Each resulting digest was subsequently measured in triplicate by F-AAS (three instrumental readings). The spectrometer was calibrated using certified standard solutions supplied by ANALYTIKA Ltd. (Prague, Czech Republic). The upper limits of the calibration ranges were 10.0 mg/L for Fe, 7.50 mg/L for Mn, 5.00 mg/L for Ni, Cu, Zn, and Pb, and 2.00 mg/L for Cd. The instrumental detection limits (IDLs) and instrumental quantification limits (IQLs) are presented in the Supplementary Materials, Table S2.

Analytical trueness was evaluated using the certified reference material BCR-414 plankton (European Commission, Joint Research Centre, Geel, Belgium), analyzed in five replicates. The material was used as an analytical quality-control sample but was not matrix-matched to bovine hair. Each analytical batch also included reagent blanks. Reference and measured values are summarized in Table S3.

Table S4 presents the mean relative standard deviation (RSD) values for triplicate instrumental measurements of the cow-hair digests. The mean RSD values ranged from 0.12% to 2.01%, indicating good repeatability of the instrumental measurements. Analytical trueness was additionally evaluated by comparing the BCR-414 results with the corresponding reference values.

Thoroughly mixed dry hair portions of 0.0400 ± 0.001 g were analyzed for total Hg using an AMA-254 direct mercury analyzer (Altec Ltd., Prague, Czech Republic). The instrument was calibrated using standards supplied by ANALYTIKA Ltd. (Prague, Czech Republic). The absolute IDL and IQL were 0.003 and 0.010 ng Hg per test portion, respectively. For a 0.0400 g test portion, these values correspond to nominal sample-basis limits of 0.000075 and 0.00025 mg/kg dw, respectively. Mercury concentrations determined in the certified reference material BCR-414 plankton are presented in Table S5.

### 2.3. Statistical Analysis

Statistical analysis was performed using GraphPad Prism version 9.2.0 (GraphPad Software, San Diego, CA, USA). Concentrations of individual metals were characterized using descriptive statistics, including the minimum, 25th percentile, median, 75th percentile, maximum, arithmetic mean with standard deviation (SD), and geometric mean. Differences in metal concentrations among animals from various districts were evaluated using the non-parametric Kruskal–Wallis test, after a non-normal data distribution was confirmed by the Shapiro–Wilk test. Multiple comparisons between groups were performed using Dunn’s post hoc test. Differences were considered statistically significant at *p* < 0.05. Moreover, correlations between metal concentrations and animal age were evaluated using Spearman’s rank correlation coefficient with the following interpretation: a coefficient value (Rs) of 0.00 to 0.19 indicated a very weak correlation, 0.20 to 0.39 a weak correlation, 0.40 to 0.69 a moderate correlation, 0.70 to 0.89 a strong correlation, and 0.90 to 1.00 a very strong correlation. Metal concentrations below the limit of detection (LOD) were imputed in the statistical analysis as LOD/2.

## 3. Results

During this study, all metals were detected in cow hair (Supplementary Materials, Table S1; Table 2). Five of the tested metals, namely Mn, Fe, Cu, Zn, and Hg, were observed in concentrations above the limit of detection (LOD) in all analyzed samples. The highest mean concentration (±standard deviation) was recorded for Fe, amounting to 274.9 ± 169.8 mg/kg of dry weight (dw) (median 248.0 mg/kg dw). A significantly lower mean concentration was found for Zn, amounting to 77.94 ± 12.91 mg/kg dw (median 79.65 mg/kg dw). Even lower mean concentrations were found for Mn and Cu, which amounted to 7.81 ± 6.06 mg/kg dw (median 6.12 mg/kg dw) and 5.63 ± 1.16 mg/kg dw (median 5.69 mg/kg dw), respectively. Among the metals whose concentrations exceeded the LOD in all analyzed samples, Hg had the lowest mean concentration, amounting to 0.0051 ± 0.0022 mg/kg dw (median 0.0049 mg/kg dw). The presence of Pb and Cd in concentrations above the LOD was found in 97.92% and 93.75% of the analyzed samples, respectively. The mean concentration of Pb reached 3.98 ± 1.47 mg/kg dw (median 3.68 mg/kg dw), while that of Cd was 0.6231 ± 0.19 mg/kg dw (median 0.60 mg/kg dw). The Ni concentration exceeded the LOD in only a small percentage of the analyzed samples (4.17%), and both its mean concentration and median were below the LOD. Data regarding the metal concentration levels are summarized in Table 2.

Only those metals found at concentration levels above the LOD in at least 50% of the samples were subjected to further analysis. Therefore, nickel was excluded from further analysis, as its levels were above the LOD in only 4.17% of the samples.

**Figure 3:**
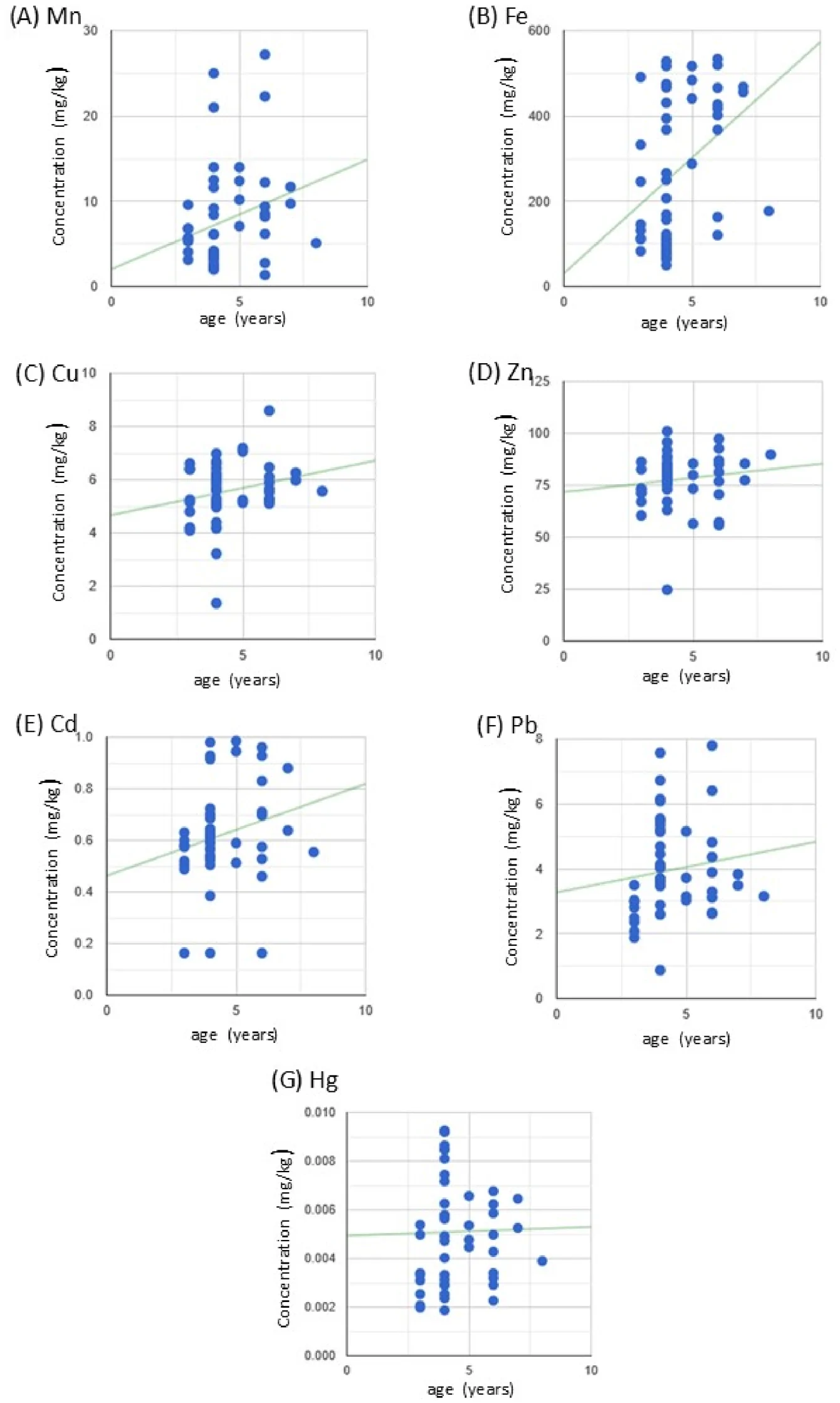
Correlation between metal concentrations in cow hair and animal age. The solid line represents the linear regression trend. The charts were generated using a publicly available Spearman correlation calculator (https://www.naukowiec.org/kalkulatory/korelacja.html, accessed on 12 September 2026).

When examining the relationships between the concentration of individual metals in the hair and animal age, a weak positive correlation was found for iron, which was statistically significant (Rs = 0.39, *p* = 0.006). For the remaining metals included in the study, Spearman’s rank correlation analysis revealed a weak or very weak positive relationship between the metal concentrations and the age of the animals; however, these relationships were statistically nonsignificant (*p* > 0.05) (Table 3, Figure 3).

**Figure 4:**
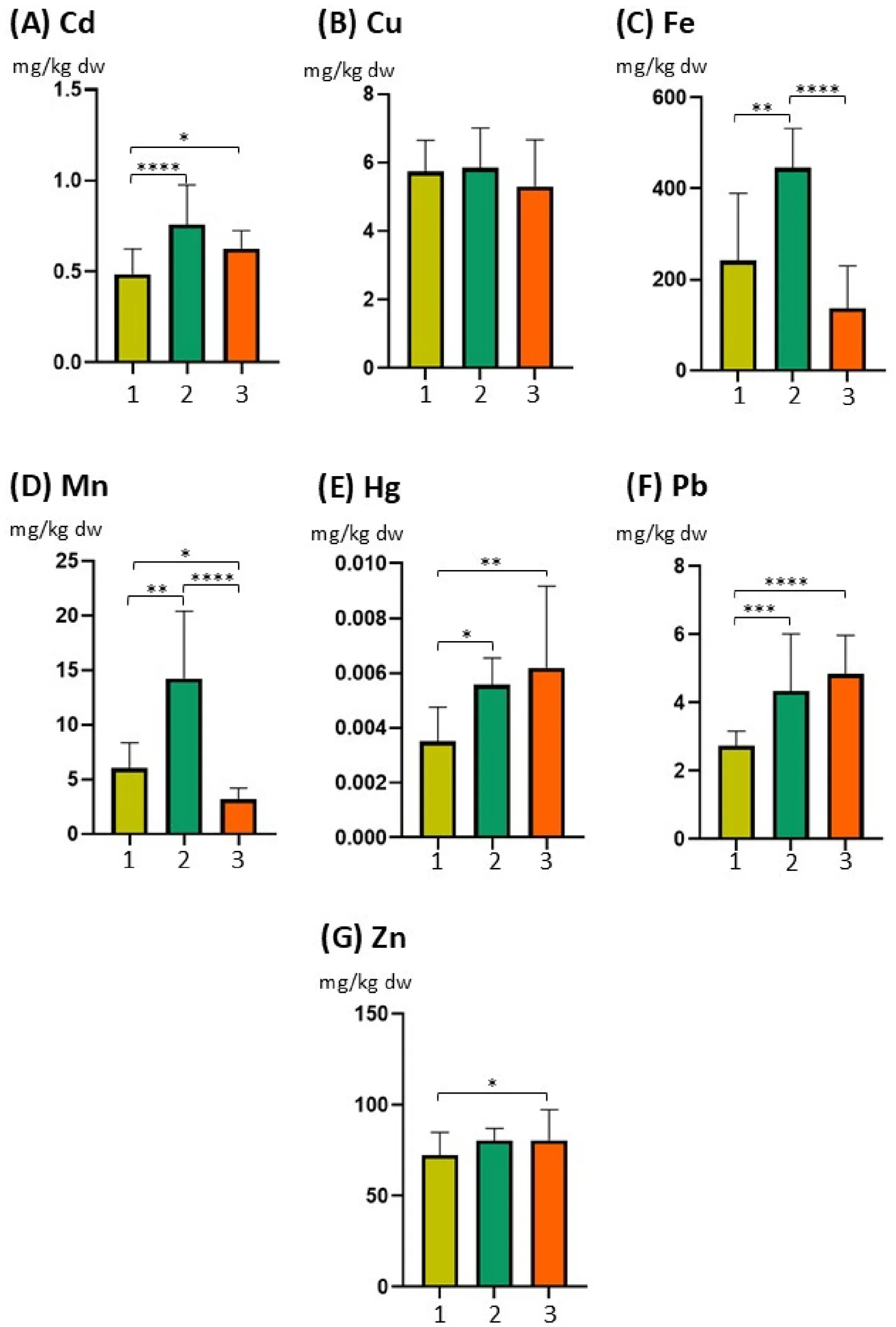
Mean metal concentrations (±standard deviation) in cow hair samples from the Sokuluk (1), Alamedin (2), and Ysyk-Ata (3) districts. Statistically significant differences are marked with asterisks: * *p* value < 0.05; ** *p* value <0.01; *** *p* value < 0.001; **** *p* value < 0.0001. The charts were generated using GraphPad Prism 9.2.0 (GraphPad Software, San Diego, CA, USA).

This study found clear differences in metal concentrations in the hair of animals raised in different districts (Figure 4; Table 4).

The highest concentrations of Cd, Fe, and Mn were found in the hair samples collected from animals in the Alamedin district. The median Cd concentration in the Alamedin district was 0.77 mg/kg (mean ± SD: 0.76 ± 0.22 mg/kg), which was statistically significantly higher (*p* < 0.0001) than that noted in the Sokuluk district (median: 0.52 mg/kg; mean: 0.49 ± 0.14 mg/kg). Cd concentrations in the Ysyk-Ata district (median: 0.61 mg/kg; mean: 0.63 ± 0.10 mg/kg) were also statistically significantly higher than those in the Sokuluk district (*p* = 0.0249). There were no statistically significant differences in Cd concentrations between the Alamedin and Ysyk-Ata districts (Figure 4A). The median concentration of Fe in the Alamedin district amounted to 466.5 mg/kg (mean: 445.4 ± 86.5 mg/kg), which was statistically significantly higher than the values observed in both the Sokuluk district, where the median was 167.0 mg/kg and the mean was 242.2 ± 147.7 mg/kg (*p* = 0.0056), and the Ysyk-Ata district (median: 99.25 mg/kg; mean: 137.1 ± 95.07 mg/kg; *p* < 0.0001). The differences in Fe concentrations between the Sokuluk and Ysyk-Ata districts were not statistically significant (*p* = 0.1205) (Figure 4C). A similar situation was noted for Mn. The median concentration of this metal in the Alamedin district was 12.3 mg/kg (mean: 14.24 ± 6.18 mg/kg), which was statistically significantly higher than the concentrations noted in the Sokuluk (median: 5.91 mg/kg; mean: 6.04 ± 2.33 mg/kg; *p* = 0.0022) and Ysyk-Ata (median: 2.99 mg/kg; mean: 3.17 ± 1.03 mg/kg; *p* < 0.0001) districts. Moreover, Mn levels noted in the Sokuluk district were statistically significantly higher than those noted in the Ysyk-Ata district (*p* = 0.0334) (Figure 4E). Hg concentrations were the highest in the Ysyk-Ata district (median: 0.0073 mg/kg; mean: 0.0062 ± 0.0029 mg/kg). However, no statistically significant differences were found (*p* > 0.9999) between these values and those observed in the Alamedin district (median: 0.0055 mg/kg; mean: 0.0056 ± 0.0010 mg/kg). The lowest Hg concentrations were found in the hair of animals from the Sokuluk district (median: 0.0033 mg/kg; mean: 0.0035 ± 0.0012 mg/kg). These values were statistically significantly lower than those observed in both the Alamedin (*p* = 0.0130) and Ysyk-Ata (*p* = 0.0098) districts (Figure 4D).

A similar situation was noted for Pb. The median concentration of this metal in the Ysyk-Ata district was 4.92 mg/kg (mean: 4.84 ± 1.13 mg/kg), and in the Alamedin district, it was 4.02 mg/kg (mean: 4.35 ± 1.66 mg/kg). There were no statistically significant differences between these values (*p* = 0.9646). The lowest Pb concentrations were found in the Sokuluk district (median: 2.72 mg/kg; mean: 2.74 ± 0.42 mg/kg), and these values were statistically significantly lower than those observed in the Alamedin (*p* = 0.0003) and Ysyk-Ata (*p* < 0.0001) districts (Figure 4F). Zn concentrations in the hair of animals from the individual districts covered by this study were more uniform. The median concentration of this metal was 83.4 mg/kg (mean: 80.68 ± 16.76 mg/kg) in the Ysyk-Ata district, 80.3 mg/kg (mean: 80.58 ± 6.49 mg/kg) in the Alamedin district, and 72.3 mg/kg (mean: 72.55 ± 12.37 mg/kg) in the Sokuluk district. Statistically significant differences were noted between the concentrations observed in the Ysyk-Ata and Sokuluk districts (*p* = 0.0273). Differences between the Sokuluk and Alamedin districts, as well as between the Alamedin and Ysyk-Ata districts, were not statistically significant (*p* = 0.2409 and *p* > 0.9999, respectively) (Figure 4G). Even more uniform concentration levels among animals from different districts were observed for Cu. The median concentration of this metal was 5.86 mg/kg (mean: 5.74 ± 0.91 mg/kg) in the Sokuluk district, 5.90 mg/kg (mean: 5.84 ± 1.16 mg/kg) in the Alamedin district, and 5.62 mg/kg (mean: 5.30 ± 1.36 mg/kg) in the Ysyk-Ata district. Differences in Cu concentrations between particular districts were not statistically significant (all *p*-values were above 0.9999) (Figure 4B). Descriptive statistics for metal concentrations in individual districts are summarized in Table 4.

No statistically significant differences were observed in the concentrations of the examined elements in hair samples obtained from different cattle breeds. Descriptive statistics for the concentrations of the studied metals in the hair of individual cattle breeds are presented in Table 5.

## 4. Discussion

These results demonstrated the widespread occurrence of most of the analyzed metals in the hair of dairy cows raised in the vicinity of Bishkek, while simultaneously confirming the utility of hair samples, as established in previous studies, for researching the exposure of livestock to elements found in the environment [30,32,33]. It should be noted that hair samples represent a unique matrix in toxicological studies, whose properties differ significantly from those of “traditional” matrices, such as blood serum or urine samples.

The fundamental difference is that substances incorporated into hair remain bound within the hair shaft, allowing the sampled hair segment to integrate exposure over its growth period [26,27]. More precisely, toxic elements bind to the sulfhydryl (-SH) groups of cysteine, forming stable complexes with metal ions [28,29]. However, it should be noted that substances can enter the hair through two pathways. The first, internal pathway involves the penetration of substances from capillaries through the hair bulb. The second, external pathway involves the penetration of substances directly from the external environment [27]. During the analysis of substance levels in hair, it is impossible to determine how much entered the hair through the internal pathway and how much through the external one. Element concentrations in hair may integrate both endogenous incorporation during hair growth and exogenous deposition of soil, dust, and airborne particles onto the hair surface. Therefore, the elemental profile of hair should be interpreted as an integrated biological–environmental signal rather than a direct measure of systemic body burden [26,28,29]. The relative contribution of both pathways may also depend on the sample-cleaning procedure [30,34]. Nevertheless, metals are released from hair only after its structure is degraded. Consequently, hair is considered a highly stable matrix. The “exposure window” examined by analyzing hair samples ranges from several weeks up to several years, depending on the species and hair length.

Therefore, the question arises as to the duration of the animals’ exposure to the metals investigated in the present study. In humans, hair grows continuously, and its analysis can reflect exposure over several years [35]. In cows, the situation is different. First, it should be noted that the hair growth rate in cows is highly variable, ranging from 0.35 to even 1.7 cm per month (with an average of 0.6 to 1 cm per month) [36,37]. This rate depends not only on the breed, animal condition, physiological state, or nutrition, but also on the ambient temperature and even the body part; it has been found, for example, that hair on the tail grows much faster than on other parts of the body [36,38]. Moreover, in cattle, the hair cycle lasts between 90 and 120 days [36,39]. Considering the reported variability in bovine hair growth and the length of the analyzed hair, the samples likely integrated exposure over a period ranging from several weeks to several months. However, the exact exposure window cannot be determined from hair length alone [36,37]. Accordingly, the analyzed hair should not be interpreted as representing exposure over the animals’ entire lifetime.

Another advantage of hair as a matrix in toxicological studies is that its collection is painless and stress-free, and samples can be easily stored and shipped over long distances. The advantages of hair as a matrix have led to the use of hair samples to assess exposure to toxic elements since the 19th century. They are utilized in studies involving both animals and humans. Regarding cows, however, the number of such studies is relatively small in comparison with observations on humans. Nevertheless, it is well known that the metal content in cow hair clearly depends on the region where the animals are raised (Table 6). This is mainly connected to the degree of urbanization and industrialization, the type of industry dominating the given area [22,40,41], as well as the local geological conditions. For example, it has been shown that animals living in volcanic areas exhibit higher levels of certain metals in their hair, such as Fe, Cu, Pb, Ni, and others [42]. Previous studies have also shown that the metal content in cow hair depends on oral exposure to these elements [43] and may be connected with the season, the number of lactations the cow has undergone, the site where the hair was sampled, or even its color, as dark hair, containing more melanin pigment, usually exhibits a higher metal content [31,32,34,44,45].

Due to a variety of factors influencing metal levels in cattle hair, a direct comparison of the results obtained in this study with previous works is difficult.

Compared with the data summarized in Table 6, Pb, Cd, and Fe concentrations were higher than those reported for several other cattle populations, whereas Cu and Zn were within the lower range of published values. Ni was detected above the LOD in only two samples; therefore, its low detection frequency should not be interpreted as evidence of a deficient Ni status. These comparisons should be considered contextual rather than diagnostic because the available studies differ in hair-sampling locations, sample-cleaning and digestion procedures, analytical techniques, and method sensitivity [26,34,45,47].

This study also revealed differences in the concentrations of the analyzed metals in the hair of cows originating from different districts. The spatial pattern was element-specific. The highest mean concentrations of Fe, Mn, and Cd were recorded in the Alamedin district, whereas Pb and Hg reached their highest mean concentrations in the Ysyk-Ata district. The lowest values were not consistently associated with a single district. Therefore, the observed distribution does not indicate a single common environmental gradient. Multiple local factors, including soil conditions, feed and water composition, atmospheric deposition, and husbandry practices, may have contributed to these elemental profiles. However, because these environmental matrices were not analyzed, their relative contributions cannot be determined from the present data [22,30,32].

It should be noted that knowledge regarding environmental pollution by harmful elements, as well as the level of exposure of humans and animals to these elements in Kyrgyzstan, is relatively limited. Nevertheless, previous studies have demonstrated the presence of the elements analyzed in this work in various environmental components and plants, as well as animal and human organisms (Table 7). Furthermore, significant variation in the concentrations of these elements has been shown depending on the sampling location, the collection period, or the specific plant parts. These results confirm that the levels of these elements in the environment and organisms depend, as mentioned above, on numerous factors.

The results obtained during this study suggest that the levels of most of the investigated metals (with the exception of Fe) do not correlate with animal age, and no statistically significant differences in element levels were observed among the different cattle breeds. These findings regarding age differ from previous observations, which noted that metal levels (including highly toxic metals) are higher in the hair of older animals [34,40,46,47]. However, it must be kept in mind that both age- and breed-related elemental profiles are heavily influenced by a variety of confounding factors, such as nutrition, milk yield, and the degree of local environmental pollution. Therefore, further comprehensive monitoring and environmental research in Kyrgyzstan are required to definitively determine whether and how these specific factors influence metal levels in the particular cattle breeds included in this study.

Moreover, because bovine hair is periodically renewed, these age-related differences should not be interpreted as lifelong metal accumulation within the same hair shaft. The mechanisms underlying the previously reported age-related differences in hair metal concentrations cannot be determined from these data.

This raises the question of whether the metal levels observed during the present study could have a negative impact on animal health and pose a potential risk to consumers of animal-derived products. To answer this question, the analyzed metals should be divided into two groups, namely those that exhibit high toxicity and are non-essential to living organisms, and those that participate in physiological processes. The first group includes Cd, Pb, and Hg; these elements perform no physiological functions in the body, and from a toxicological point of view, the lower their concentration in tissues, the better. However, it should be noted that their presence in various tissues may result from geogenic sources and baseline environmental background exposure, and their current low concentrations do not exceed established toxicity thresholds. No legally binding or universally accepted toxicological thresholds have been established for Cd, Pb, or Hg concentrations in bovine hair. Published background or reference ranges may provide comparative information, but these values vary considerably among regions, animal populations, and analytical procedures. Cd and Pb concentrations were higher than some published comparative values, whereas Hg concentrations were lower. However, hair concentrations alone cannot be used to diagnose toxic effects or definitively determine the health status of the animals [26,43,46,50]. It cannot be assumed that the toxic metal levels recorded in this study are completely neutral to the animals. Previous studies have demonstrated that even cow exposure to relatively low levels of toxic metals, especially long-term exposure, causes subclinical toxicity and can negatively affect animal health, resulting, among others, in reproductive cycle disorders, impaired immunity, and internal organ dysfunction [31,51,52,53]. These observations suggest that the levels of toxic metals observed during this work may have an impact on animal health. However, it should be emphasized that these are only assumptions, and a definitive determination of the effects of the observed concentrations on animal health requires further comprehensive clinical and toxicological studies.

Another important aspect of cattle exposure to toxic metals is the concentration of these metals in products of animal origin. Previous studies have demonstrated a clear positive correlation between Pb concentrations in the hair and milk of cows, whereas no such correlations were found for Hg and Cd [33,43,54]. This lack of correlation may result from the fact that the mammary gland acts as a barrier to Hg and Cd, which is supported by studies where Cd was administered orally, yet its levels in milk remained low, in contrast to the levels observed in hair [43]. Furthermore, previous research has shown that the levels of toxic metals in hair often deviate from those observed in milk [33,41,55,56]. This discrepancy may be related to the fact that hair is a matrix integrating exposure to environmental contaminants over the hair-growth period. Although relationships between metal concentrations in cow hair and milk have been reported, these associations are element-specific and are not consistently observed for Cd, Pb, and Hg [33,43,54]. Because milk was not analyzed, the obtained results cannot be used to assess metal concentrations in dairy products or the associated risk to consumers. Instead, they provide a strong rationale for future paired analyses of hair, milk, feed, and drinking water.

The situation is different for metals such as Cu, Fe, Mn, and Zn, which are microelements involved in various physiological processes, and their presence in the body is essential for proper functioning. In their case, two aspects related to animal health must be considered: excessive concentration, which can result in toxicity, and deficiency, which leads to impaired bodily functions.

The reference ranges proposed by Puls [57], which in cow hair dry matter amount to 6.7–32 mg/kg for Cu, 59–200 mg/kg for Fe, 0.5–1.32 mg/kg for Mn, and 100–150 mg/kg for Zn, remain a frequently used historical benchmark in veterinary mineral assessment. However, they should be interpreted as comparative ranges rather than universal diagnostic or toxicological thresholds for bovine hair. Contemporary assessment of trace-element status in ruminants requires the combined evaluation of dietary sources and appropriate biological matrices, as hair concentrations may be affected by age, hair color, sampling location, and sample-preparation procedures [47,57,58].

Comparing the results obtained in this work with the reference data described by Puls, it can be concluded that the Fe and Mn concentrations recorded in many Kyrgyz cows exceeded these reference limits. In the case of Mn, however, the observed values did not exceed the level established by Puls as “high/toxic” (>60 mg/kg) [57]. The situation was different for Fe, whose concentrations in some samples, particularly those collected in the Alamedin district, exceeded the comparative ranges reported by Puls [57]. This finding indicates an elevated Fe content in the hair but does not determine its origin. Hair analysis alone cannot distinguish among increased dietary intake, systemic accumulation, external deposition of Fe-containing particles, or a combination of these processes. Consequently, the obtained values cannot be considered direct evidence of chronic Fe overload, oxidative stress, or systemic toxicity without supporting measurements in feed, water, blood, or tissues [58,59].

High dietary Fe may influence the bioavailability of other trace elements. However, this mechanism was not directly evaluated in the present study, and the observed district-level data do not indicate a consistent inverse relationship between Fe and Cu or Zn. In the Alamedin district, the highest mean Fe concentration coincided with the highest mean Cu concentration, whereas the mean Zn concentration was similar to that recorded in Ysyk-Ata. Moreover, Ni was detected above the LOD in only two samples. Therefore, Fe-induced deficiencies of Cu, Zn, or Ni should be considered a hypothesis requiring confirmation through analyses of feed, drinking water, blood, or liver rather than a mechanism demonstrated by the current hair data [58,60].

This study has certain limitations. The most significant of these is the lack of analysis of the levels of the investigated elements in the feed and drinking water used for the animals, as well as in the farm environment (e.g., in dust or pasture plants). Without such comprehensive research, it is impossible to determine the main sources of exposure to the analyzed elements for the animals included in the study. Further limitations of this study include the relatively small sample size, the uneven distribution of animals across individual breeds, the lack of precise data on individual milk production levels, and the absence of hematological analyses. Despite these constraints, this study provides the first evaluation of selected element levels in the hair of dairy cows in the Bishkek region. Thereby, it contributes to the development of knowledge on livestock exposure to these elements in Kyrgyzstan and can serve as a baseline for future comprehensive research on this topic.

## 5. Conclusions

This study demonstrated the occurrence and spatial variability of selected trace elements and potentially toxic metals in the hair of cattle reared in the Bishkek area. Fe showed the highest overall concentration; Pb and Cd were detected in most samples, whereas Ni was detected above the LOD in only two samples. The elemental pattern differed among districts: Alamedin showed the highest mean concentrations of Fe, Mn, and Cd, whereas Ysyk-Ata showed the highest mean concentrations of Pb and Hg; Cu and Zn were more evenly distributed. Compared with literature data, Pb, Cd, and Fe concentrations were relatively high, whereas Cu and Zn were within the lower range of published values. However, cross-study comparisons should be interpreted cautiously due to methodological differences. The element-specific spatial pattern does not allow the observed concentrations to be attributed to a single environmental source and does not provide evidence of Fe-induced Cu, Zn, or Ni deficiencies. Moreover, hair data alone cannot determine the effects of these elements on animal health or their concentrations in milk.

## Figures and Tables

**Table 1:** Characterization of the districts included in this study.

Region Name	Sokuluk	Alamedin	Ysyk-Ata
Area (km^2^)	2550	1503	2415
Human population in 2024 (in thousands)	250.6	180.6	158.1
Human population density (thousands/km^2^)	0.098	0.120	0.065
Administrative center	Sokuluk	Lebedinovka	Kant
Distance in a straight line from the center of Bishkek to the administrative center of the region (km)	22.29	9.50	20.0
Estimated annual mean PM2.5 concentrations (µg/m^3^)	22–30	35–45	18–23
Main industry	Agri-food processing	Energy and commuter services	Heavy industry (mining and construction materials industries)
Number of official landfills	18	12	4
Landfill area (ha)	14.8	8.5 *	19.3

* Most of the waste is transported to an authorized landfill in the city of Bishkek.

**Table 2:** Levels (mg/kg dw) and frequency (%) of metal detection in cow hair samples (*n* = 48)—cumulative data.

	Mn	Fe	Ni	Cu	Zn	Cd	Pb	Hg
Minimum	1.35	50.2	<LOD	1.36	24.70	<LOD	<LOD	0.0019
25th percentile	3.39	111.5	<LOD	5.12	73.10	0.53	3.00	0.0031
Median	6.12	248.0	<LOD	5.69	79.65	0.60	3.68	0.0049
75th percentile	10.08	452.3	<LOD	6.37	85.50	0.70	5.06	0.0065
Maximum	27.20	534.0	2.48	8.59	101.00	0.99	7.80	0.0093
Mean	7.81	274.9	<LOD	5.63	77.94	0.62	3.98	0.0051
SD	6.06	169.8	-	1.16	12.91	0.19	1.47	0.0022
Geometric mean	6.04	217.1	<LOD	5.47	76.50	0.59	3.71	0.0046
% samples > LOD	100	100	4.17	100	100	93.75	97.92	100

**Table 3:** Correlations between metal concentrations (mg/kg dw) and animal age.

	Mn	Fe	Cu	Zn	Cd	Pb	Hg
Rs	0.259	0.39	0.221	0.13	0.226	0.129	0.02
*p*	0.076	0.006	0.131	0.38	0.123	0.381	0.894
Degree of correlation	week	week	week	every week	week	every week	week

**Table 4:** Concentrations of metals (mg/kg dw) in cow hair samples from the Sokuluk, Alamedin, and Ysyk-Ata districts (*n* = 16 per district).

Metal	District	Min.	25th Perc.	Median	75th Perc.	Max.	Mean (±SD)	Geometric Mean
Cd	Sokuluk	<LOD	0.47	0.52	0.58	0.63	0.49 (0.14)	0.46
	Alamedin	<LOD	0.64	0.77	0.94	0.99	0.76 (0.22)	0.71
	Ysyk Ata	0.52	0.55	0.61	0.67	0.92	0.63 (0.10)	0.62
Cu	Sokuluk	4.09	5.12	5.86	6.46	7.07	5.74 (0.91)	5.67
	Alamedin	3.22	5.16	5.90	6.22	8.59	5.84 (1.16)	5.73
	Ysyk Ata	1.36	4.54	5.62	6.12	7.30	5.30 (1.36)	5.04
Fe	Sokuluk	82.80	115.0	167.0	368.0	517.0	242.2 (147.7)	203.1
	Alamedin	207.0	428.0	466.5	508.8	534.0	445.4 (86.50)	434.8
	Ysyk Ata	50.20	77.30	99.25	167.5	394.0	137.1 (93.07)	115.8
Hg	Sokuluk	0.0020	0.0026	0.0033	0.0040	0.0066	0.0035 (0.0012)	0.0033
	Alamedin	0.0043	0.0048	0.0055	0.0061	0.0081	0.0056 (0.0010)	0.0055
	Ysyk Ata	0.0019	0.0029	0.0073	0.0091	0.0093	0.0062 (0.0029)	0.0053
Mn	Sokuluk	1.35	4.32	5.91	7.86	10.20	6.04 (2.33)	5.50
	Alamedin	7.07	9.47	12.30	19.25	27.20	14.24 (6.18)	13.16
	Ysyk Ata	1.99	2.35	2.99	3.63	6.13	3.17 (1.03)	3.04
Pb	Sokuluk	1.88	2.51	2.72	3.10	3.50	2.74 (0.42)	2.71
	Alamedin	<LOD	3.54	4.02	5.07	7.80	4.35 (1.66)	3.98
	Ysyk Ata	3.47	3.68	4.92	5.95	6.73	4.84 (1.13)	4.72
Zn	Sokuluk	55.80	61.08	72.30	81.45	97.40	72.55 (12.37)	71.58
	Alamedin	70.70	75.68	80.30	85.43	95.80	80.58 (6.49)	80.34
	Ysyk Ata	24.70	79.10	83.40	88.78	101.0	80.68 (16.76)	77.86

**Table 5:** Concentrations of metals (mg/kg dw) in hair samples from cows of the Alatau (*n* = 13), Kyrgyz (*n* = 16), and Swiss (*n* = 16) breeds.

Metal	Breed	Min.	25th Perc.	Median	75th Perc.	Max.	Mean (±SD)	Geometric Mean
Cd	Alatau	0.505	0.54	0.63	0.71	0.985	0.66 (0.14)	0.64
	Kyrgyz	<LOD	0.53	0.59	0.70	0.96	0.58 (0.20)	0.53
	Swiss	<LOD	0.52	0.59	0.92	1.00	0.65 (0.22)	0.61
Cu	Alatau	4.20	5.25	5.98	6.28	7.07	5.81 (0.74)	5.76
	Kyrgyz	4.20	5.23	5.69	6.45	8.59	5.93 (1.03)	5.85
	Swiss	1.36	4.40	5.18	6.25	7.19	5.26 (1.42)	4.99
Fe	Alatau	66.10	102.6	163.0	471.5	517.0	253.2 (179.0)	196.3
	Kyrgyz	64.40	123.8	258.0	463.5	534.0	290.5 (173.9)	234.1
	Swiss	50.20	111.0	333.0	431.0	528.0	276.6 (167.8)	218.2
Hg	Alatau	0.0020	0.0035	0.0050	0.0064	0.0092	0.0051 (0.0020)	0.0047
	Kyrgyz	0.0019	0.0030	0.0036	0.0064	0.0093	0.0047 (0.0023)	0.0042
	Swiss	0.0020	0.0033	0.0054	0.0081	0.0092	0.0055 (0.0024)	0.0049
Mn	Alatau	2.31	3.15	5.71	10.90	21.00	7.22 (5.28)	5.82
	Kyrgyz	1.35	3.46	6.15	9.70	27.20	8.19 (7.19)	6.01
	Swiss	2.24	3.44	7.51	12.43	25.00	8.20 (5.82)	6.54
Pb	Alatau	2.48	2.85	3.55	5.85	7.57	4.27 (1.72)	3.97
	Kyrgyz	1.88	2.77	3.59	4.28	7.80	3.86 (1.55)	3.61
	Swiss	<LOD	3.03	3.72	5.14	6.08	3.87 (1.26)	3.61
Zn	Alatau	56.60	74.35	79.90	88.55	95.80	79.45 (11.16)	78.68
	Kyrgyz	55.80	70.83	78.05	86.70	101,0	77.58 (12.07)	76.66
	Swiss	24.70	73.40	80.70	85.50	97.40	77.20 (15.12)	74.92

**Table 6:** Mean metal concentrations (mg/kg dw ± SD) observed in the present work compared to reported values from various regions of the world.

Element.	Country and Mean Concentration (±SD)	Reference
Cd	**Albania:** 2.36 ± 1.37 and 2.00 ± 0.79 (depending on localization)	[22]
	**Croatia:** 0.014 ± 0.002 (in a gas industry region)	[41]
	**India**: from 0.52 ± 0.06 to 2.62 ± 0.32 (depending on localization)	[40]
	**Italy:** 0.03 ± 0.03	[34]
	**Korea:** from 0.04 ± 0.006 to 0.02 ± 0.002 (depending on reproductive status)	[31]
	**Poland:** from 0.03 ± 0.006 to 0.07 ± 0.001 (depending on localization and season)	[32]
	**Russia:** 0.004 ± 0.002	[46]
Cu	**Albania:** 17.84 ± 7.75 and 15.84 ± 4.48 (depending on localization)	[22]
	**Croatia**: 4.77 ± 0.33 (in a gas industry region)	[41]
	**Italy:** 4.92 ± 2.2	[34]
	**Korea:** from 9.75 ± 1.65 to 9.65 ± 0.65 (depending on reproductive status)	[31]
	**Poland:** from 10.16 ± 5.65 to 32.04 ± 3.29 (depending on localization and season)	[32]
	**Russia**: 8.71 ± 0.979	[46]
Fe	**Albania:** 463 ± 168 and 4.37 ± 133 (depending on localization)	[22]
	**Italy:** 175.11 ± 143.39	[34]
	**Korea:** 357.03 ± 73.82 and 140.41 ± 30.1 (depending on reproductive status)	[31]
	**Poland:** from 58.25 ± 10.73 to 237.45 ± 46.07 (depending on localization and season)	[32]
	**Russia:** 199.6 ± 211.2	[46]
Hg	**Croatia:** 0.0061 ± 0.0008 (in a gas industry region)	[41]
	**Korea**: from 0.045 ± 0.01 to 0.15 ± 0.08 (depending on reproductive status)	[31]
	**Russia:** 0.004 ± 0.003	[46]
Mn	**Albania:** 6.20 ± 2.72 and 5.63 ± 1.52 (depending on localization)	[22]
	**Korea:** from 42.12 ± 4.42 to 30.69 ± 4.69 (depending on reproductive status)	[31]
	**Poland:** from 3.75 ± 0.92 to 20.02 ±4.74 (depending on localization and season)	[32]
	**Russia:** 5.18 ± 2.4	[46]
Ni	**Albania:** 3.42 ± 1.73 and 2.96 ± 0.98 (depending on localization)	[22]
	**Italy:** 3.73 ± 4.34	[34]
	**Korea:** from 0.39 ± 0.05 to 0.28 ± 0.03 (depending on reproductive status)	[31]
	**Russia:** 0.211 ± 0.120	[46]
Pb	**Albania:** 3.02 ± 1.63 and 2.50 ± 1.03 (depending on localization)	[22]
	**Croatia:** 0.609 ± 0.153 (in a gas industry region)	[41]
	**India:** from 2.99 ± 0.27 to 15.09 ± 70.85 (depending on localization)	[40]
	**Italy:** 0.97 ± 1.39	[34]
	**Korea:** from 1.975 ± 0.53 to 1.25 ± 0.15 (depending on reproductive status)	[31]
	**Poland:** from 0.35 ± 0.12 to 2.34 ± 0.57 (depending on localization and season)	[32]
	**Russia:** 0.094 ± 0.063	[46]
Zn	**Albania:** 162 ± 57 and 150 ± 35 (depending on localization)	[22]
	**Croatia:** 115 ± 7 (in a gas industry region)	[41]
	**Italy:** 54.43 ± 19.34	[34]
	**Korea:** from 510.02 ± 112.6 to 550.52 ± 68.43 (depending on reproductive status)	[31]
	**Poland:** from 125.66 ± 19.3 to 427.38 ± 54.24 (depending on localization and season)	[32]
	**Russia:** 27.33 ± 16.39	[46]

**Table 7:** Concentrations of the analyzed elements (mean ± SD or median) in the environment and living organisms in Kyrgyzstan, as reported in previous studies. Concentrations are presented in mg/kg, except for water and mare follicular fluid samples, where the units are µg/L.

Matrix	Concentration Levels	References
River water	Depending on the water flow volume (season). **Cu:** 3.64 ± 2.29 (3.54) and 4.23 ± 3.62 (3.09); **Pb:** 1.01 ± 0.641 (0.9) and 1.52 ± 1.26 (1.32); **Zn:** 5.43 ± 2.13 (5.06) and 9.60 ± 6.79 (8.2)	[6]
Soil	Depending on the region. **Cd:** from 2.182 ± 0.019 to 4.106 ± 0.018; **Cu:** from 46.49 ± 0.258 to 122.447 ± 1.207; **Fe:** from 5499.519 ± 36.277 to 8646.888 ± 43.034; **Mn:** from 110.518 ± 0.888 to 216.574 ± 0.756; **Ni:** from 9.925 ± 0.077 to 30.917 ± 0.089; **Pb:** from 16.59 ± 0.088 to 51.148 ± 0.159; **Zn:** from 223.505 ± 1.560 to 339.619 ± 1.428	[48]
Soil	Depending on the region. **Cu:** 0.68 and 6.8; **Hg:** 0.05 and 0.1; **Pb:** 0.1 and 0.3; **Zn:** 1.05 and 5.0	[20]
Soil	Depending on the location of the sampling place. **Cu:** from 47.136 ± 1.395 to 324.366 ± 3.092; **Fe:** from 5986.692 ± 147.311 to 8281.466 ± 63.051; **Ni:** from 48.164 ± 0.869 to 84.241 ± 1.913; **Pb:** from 40.66 ± 0.641 to 414.466 ± 7.804; **Zn:** from 177.298 ± 3.647 to 345.098 ± 6.473	[9]
Soil from mining areas	Depending on localization. **Cd:** from 1.5 to 15; **Cu:** from 25 to 110; **Hg:** from 0.2 to 1.5; **Pb:** from 75 to 450; **Zn:** from 120 to 820	[3]
Leafy vegetables	Depending on the region and vegetable species. **Cd:** from 0.01432 ± 0.00028 to 0.09152 ± 0.00136; **Cu:** from 5.935 ± 0.062 to 65.045 ± 0.630; **Fe:** from 141.113 ± 2.48 to 789.539 ± 4.35; **Mn:** from 15.271 ± 0.355 to 158.201 ± 1.54; **Ni:** from 0.090684 ± 0.00047 to 1.30035 ± 0.00977; **Pb:** from 0.001862 ± 0.000007 to 0.010079 ± 0.00014; **Zn:** from 28.417 ± 0.35 to 327.818 ± 5.3	[7]
Fruity vegetables	Depending on the region and vegetable species. **Cd** from 0.007514 ± 0.000061 to 0.088763 ± 0.000871; **Cu:** from 0.324 ± 0.03 to 8.555 ± 0.065: **Fe:** from 4.354 ± 0.048 to 130.716 ± 1.295; **Mn:** from 1.08 ± 0.017 to 81.309 ± 0.785; **Ni:** from 0.038828 ± 0.000375 to 1.252507 ± 0.012557; **Zn:** from 1.627 ± 0.006 to 89.72 ± 0.889; **Pb:** from 0.000038 ± 0.000009 to 0.002958 ± 0.00002	[8]
Pasture plants	Depending on the region. **Cu:** 7.1 and 8.5; **Hg:** 0.08 and 0.05; **Ni:** 0.99 and 2.0; **Pb:** 1.07 and 3.0; **Zn:** 26.5 and 30.0	[20]
Common walnut (*Juglans regia*)	Depending on the region. **Cu:** from 12.27 ± 0.15 to 20.67 ± 0.27; **Fe:** from 33.56 ± 0.67 to 48.92 ± 0.66; **Mn:** from 30.45 ± 0.47 to 45.61 ± 0.93; **Zn:** from 23.20 ± 0.43 to 34.63 ± 0.43	[48]
red cedar (*Juniperus* *virginiana*)	Depending on the plant part and the Bishkek district. **Cd:** from 1.278 ± 0.023 to 7.53 ± 0.082; **Cu:** from 10.500 ± 0.144 to 66.380 ± 0.720; **Fe** from 136.965 ± 1.154 to 7082.566 ± 76.833: **Mn:** from 11.287 ± 0.237 to 314.397 ± 3.410; **Pb:** from 11.934 ± 0.211 to 129.484 ± 1.404; **Zn:** from 17.534 ± 0.204 to 381.125 ± 4.134	[49]
Black poplar (*Populus nigra*)	Depending on plant part and localization. **Cu:** from 0.383 ± 0.006 to 14.134 ± 0.227, **Fe:** from 3.551 ± 0.062 to 1679.544 ± 27.231; **Ni:** from 0.19 ± 0.005 to 12.683 ± 0.229; **Pb** from 0.321 ± 0.001 to 49.466 ± 0.27; **Zn:** from 7.95 ± 0.031 to 91.594 ± 2.259	[9]
Crack willow (*Salix fragilis*)	Depending on the plant part and localization. **Cu:** from 1.795 ± 0.046 to 14.167 ± 0.28; **Fe:** from 21.045 ± 0.614 to 327.21 ± 7.843; **Ni:** from 0.477 ± 0.013 to 15.647 ± 0.463; **Pb:** from 3.379 ± 0.105 to 196.799 ± 3.657; **Zn:** from 6.63 ± 0.151 to 218.522 ± 1.392	[9]
Mare follicular fluid	**Cd:** 3.0; **Hg:** 5.0; **Pb:** 2.0; **Ni:** 5.0	[19]
Cow whole blood	Depending on the region. **Cu:** 54.91 and 89.91; **Hg:** 0.00047 and 0.0047; **Ni:** 0.047 and 0.113; **Pb:** 0.028 and 0.094; **Zn:** 296.42 and 377.36	[20]
Human hair	**Cd:** 0.12; **Hg:** 0.23; **Pb:** 8.86	[17]
Human whole blood	Depending on the region and the degree of environmental pollution. **Cd:** 0.340 and 0.320; **Cu:** 1371 and 1478; **Fe:** 417 and 366; **Hg:** 0.420 and 0.100; **Mn:** 0.820 and 0.980; **Pb:** 12.4 and 12.2; **Zn:** 6145 and 5893	[18]

## Data Availability

All data are available in the manuscript and Supplementary Materials.

## Supplementary Materials

The following supporting information can be downloaded at https://www.mdpi.com/article/10.3390/ani16213443/s1. Table S1: Animals included in the study and element levels (mg/kg) in individual hair samples; Table S2: Instrumental detection and quantification limits for the iCE 3500 spectrometer (mg/L) (Thermo Fisher Scientific, 2011 [61]); Table S3: Comparison of reference and measured element mass fractions in BCR-414 plankton determined by F-AAS (mg/kg dw; *n* = 5); Table S4: Mean RSD values (%) for triplicate instrumental measurements of cow-hair digests by F-AAS; Table S5: Comparison of certified and measured Hg mass fractions in BCR-414 plankton (mg/kg dw; *n* = 5).
