# Supplementary material for: Selected Microelements and Toxic Metals in the Hair of Dairy Cows from the Bishkek Area, Kyrgyzstan
Source: Animals (Basel). 2026 Oct 31;16(21):3443. doi: 10.3390/ani16213443 (PMC13762142; doi:10.3390/ani16213443)
Supplement: Supplementary file 1 [file animals-16-03443-s001.zip › animals-4633420-supplementary.pdf]

Supplementary materials to

# Selected microelements and toxic metals in the hair of dairy cows from the Bishkek area, Kyrgyzstan

**Slawomir Gonkowski [1,*], Paweł Świsłowski [2], Nariste Kadyralieva [3], Liliana Rytel [4] and Małgorzata Rajfur [2]**

1 Department of Clinical Physiology, Faculty of Veterinary Medicine, University of Warmia and Mazury in Olsztyn, Oczapowskiego 13, 10-957 Olsztyn, Poland
2 Institute of Biology, University of Opole, Kominka 6a, 45-032 Opole, Poland; pawel.swislowski@uni.opole.pl (P.Ś.); rajfur@uni.opole.pl (M.R.)
3 Department of Histology and Embryology, Veterinary Faculty, Kyrgyz-Turkish Manas University,
720044 Bishkek, Kyrgyzstan; nariste.kadyralieva@manas.edu.kg
4 Department of Internal Diseases with Clinics, Faculty of Veterinary Medicine, University of Warmia and Mazury in Olsztyn, Oczapowskiego 14, 10-957 Olsztyn, Poland; liliana.rytel@uwm.edu.pl
* Correspondence: slawomir.gonkowski@uwm.edu.pl

**Table S1 Animals included in the study and element levels (mg/kg) in individual hair samples**

| № | Breed | Age (years) | Mn | Fe | Ni | Cu | Zn | Cd | Pb | Hg |
|---|---|---|---|---|---|---|---|---|---|---|
| Sokuluk district | | | | | | | | | | |
| 1 | Alatau | 3 | 5,31 | 113 | < 1.25 | 4,20 | 82,7 | 0,505 | 2,81 | 0,001977 |
| 2 | Alatau | 4 | 4,07 | 110 | < 1.25 | 6,43 | 63,1 | 0,505 | 2,88 | 0,002901 |
| 3 | Kyrgyz | 3 | 4,06 | 132 | < 1.25 | 6,61 | 73,1 | 0,575 | 1,88 | 0,003089 |
| 4 | Kyrgyz | 8 | 5,08 | 177 | < 1.25 | 5,57 | 89,8 | 0,555 | 3,15 | 0,003901 |
| 5 | Alatau | 5 | 10,2 | 517 | < 1.25 | 7,07 | 56,6 | 0,513 | 3,14 | 0,006574 |
| 6 | Alatau | 4 | 6,11 | 157 | < 1.25 | 6,15 | 77,7 | 0,590 | 2,60 | 0,004025 |
| 7 | Kyrgyz | 6 | 1,35 | 121 | < 1.25 | 5,11 | 57,3 | 0,460 | 2,64 | 0,002266 |
| 8 | Swiss | 4 | 8,41 | 368 | < 1.25 | 6,60 | 77,2 | 0,385 | 2,60 | 0,003328 |
| 9 | Kyrgyz | 6 | 6,17 | 402 | < 1.25 | 6,47 | 55,8 | < 0.325 | 2,61 | 0,003202 |
| 10 | Alatau | 3 | 5,71 | 246 | < 1.25 | 6,41 | 73,4 | 0,630 | 2,48 | 0,004976 |
| 11 | Swiss | 3 | 6,82 | 333 | < 1.25 | 4,80 | 86,4 | 0,583 | 2,36 | 0,00538 |
| 12 | Swiss | 6 | 8,20 | 368 | < 1.25 | 5,53 | 97,4 | 0,528 | 3,12 | 0,003393 |
| 13 | Swiss | 3 | 3,14 | 111 | < 1.25 | 4,09 | 60,4 | 0,520 | 2,99 | 0,002538 |
| 14 | Kyrgyz | 3 | 6,82 | 82,8 | < 1.25 | 5,24 | 71,5 | < 0.325 | 2,07 | 0,003329 |
| 15 | Kyrgyz | 3 | 9,60 | 491 | < 1.25 | 6,40 | 71,2 | 0,600 | 3,50 | 0,003393 |
| 16 | Swiss | 3 | 5,54 | 146 | < 1.25 | 5,15 | 67,2 | 0,488 | 3,03 | 0,002071 |
| Alamedin district | | | | | | | | | | |
| 1 | Swiss | 4 | 9,16 | 207 | < 1.25 | 3,22 | 73,1 | < 0.325 | < 1.75 | 0,005624 |
| 2 | Alatau | 4 | 11,6 | 475 | < 1.25 | 5,89 | 95,8 | 0,688 | 7,57 | 0,004718 |
| 3 | Kyrgyz | 7 | 9,73 | 456 | < 1.25 | 6,26 | 85,5 | 0,638 | 3,49 | 0,006458 |
| 4 | Swiss | 4 | 12,5 | 431 | < 1.25 | 5,11 | 80,7 | 0,980 | 4,14 | 0,005789 |
| 5 | Swiss | 4 | 14,0 | 467 | < 1.25 | 5,99 | 76,8 | 0,928 | 3,67 | 0,008102 |
| 6 | Swiss | 4 | 25,0 | 528 | 1,35 | 6,98 | 84,6 | 0,570 | 4,46 | 0,005745 |
| 7 | Kyrgyz | 6 | 8,53 | 417 | < 1.25 | 5,66 | 81,4 | 0,960 | 3,89 | 0,005856 |
| 8 | Kyrgyz | 6 | 22,3 | 520 | < 1.25 | 8,59 | 87,1 | 0,698 | 4,36 | 0,006236 |
| 9 | Alatau | 4 | 21,0 | 517 | < 1.25 | 5,04 | 75,3 | 0,643 | 5,48 | 0,004922 |
| 10 | Swiss | 6 | 9,38 | 427 | < 1.25 | 6,10 | 76,9 | 0,928 | 3,29 | 0,004275 |
| 11 | Kyrgyz | 6 | 12,2 | 466 | < 1.25 | 5,23 | 85,2 | 0,710 | 7,80 | 0,006772 |
| 12 | Swiss | 5 | 14,0 | 441 | < 1.25 | 7,19 | 85,5 | 0,945 | 3,72 | 0,004464 |
| 13 | Alatau | 7 | 11,7 | 468 | < 1.25 | 5,98 | 77,4 | 0,880 | 3,83 | 0,005256 |
| 14 | Swiss | 5 | 12,4 | 484 | < 1.25 | 5,14 | 73,4 | 0,590 | 5,15 | 0,004771 |
| 15 | Alatau | 5 | 7,07 | 288 | < 1.25 | 5,23 | 79,9 | 0,985 | 3,03 | 0,005366 |
| 16 | Kyrgyz | 6 | 27,2 | 534 | 2,48 | 5,90 | 70,7 | 0,830 | 4,82 | 0,004973 |
| Ysyk Ata district | | | | | | | | | | |
| 1 | Kyrgyz | 4 | 3,35 | 99,1 | < 1.25 | 5,71 | 88,4 | 0,540 | 3,47 | 0,009254 |
| 2 | Swiss | 4 | 2,47 | 123 | < 1.25 | 4,40 | 83,7 | 0,915 | 4,03 | 0,002355 |
| 3 | Alatau | 4 | 2,31 | 66,1 | < 1.25 | 5,59 | 81,2 | 0,723 | 3,55 | 0,007444 |
| 4 | Swiss | 4 | 3,66 | 113 | < 1.25 | 6,25 | 79,0 | 0,628 | 4,69 | 0,007173 |
| 5 | Alatau | 4 | 2,53 | 76,2 | < 1.25 | 6,04 | 91,9 | 0,685 | 5,54 | 0,006258 |
| 6 | Swiss | 4 | 3,24 | 80,6 | < 1.25 | 5,90 | 83,1 | 0,623 | 6,08 | 0,009225 |
| 7 | Alatau | 4 | 3,55 | 95,1 | < 1.25 | 6,15 | 85,2 | 0,613 | 6,15 | 0,00924 |
| 8 | Swiss | 4 | 3,50 | 99,4 | < 1.25 | 6,68 | 88,9 | 0,563 | 3,67 | 0,009209 |
| 9 | Swiss | 4 | 2,29 | 85,1 | < 1.25 | 1,36 | 24,7 | 0,523 | 5,14 | 0,008476 |
| 10 | Kyrgyz | 4 | 6,13 | 266 | < 1.25 | 5,64 | 76,7 | 0,540 | 6,73 | 0,002933 |
| 11 | Swiss | 4 | 4,20 | 394 | < 1.25 | 4,18 | 85,9 | 0,593 | 5,20 | 0,003129 |
| 12 | Alatau | 6 | 2,75 | 163 | < 1.25 | 5,28 | 92,7 | 0,575 | 6,41 | 0,002904 |
| 13 | Kyrgyz | 4 | 1,99 | 169 | < 1.25 | 4,97 | 79,4 | 0,533 | 3,69 | 0,00187 |
| 14 | Kyrgyz | 4 | 2,69 | 64,4 | < 1.25 | 7,30 | 101 | 0,703 | 3,68 | 0,008473 |
| 15 | Swiss | 4 | 2,24 | 50,2 | < 1.25 | 5,18 | 81,9 | 0,643 | 5,35 | 0,008652 |
| 16 | Kyrgyz | 4 | 3,77 | 250 | < 1.25 | 4,20 | 67,1 | 0,610 | 4,05 | 0,002536 |

**Table S2. Instrumental detection and quantification limits for the iCE 3500 spectrometer (mg/L) (Thermo Fisher Scientific, 2011 [61]).**

| Element | IDL | IQL |
|---|---|---|
| Mn | 0.0016 | 0.020 |
| Fe | 0.0043 | 0.050 |
| Ni | 0.0043 | 0.050 |
| Cu | 0.0045 | 0.033 |
| Zn | 0.0033 | 0.010 |
| Cd | 0.0028 | 0.013 |
| Pb | 0.0130 | 0.070 |

IDL, instrumental detection limit; IQL, instrumental quantification limit.

**Table S3. Comparison of reference and measured element mass fractions in BCR-414 plankton determined by F-AAS (mg/kg dw; n = 5).**

| Element | Reference value | Measured value | Relative difference (%) |
|---|---|---|---|
| Mn | 299 ± 13 | 284 ± 13 | −5.0 |
| Fe | 1850 ± 190[a] | 1790 ± 200 | −3.2 |
| Ni | 18.8 ± 0.8 | 18.2 ± 0.9 | −3.2 |
| Cu | 29.5 ± 1.3 | 28.4 ± 1.6 | −3.7 |
| Zn | 111.6 ± 2.5 | 107 ± 3 | −4.5[b] |
| Cd | 0.383 ± 0.014 | 0.371 ± 0.018 | −3.1 |
| Pb | 3.97 ± 0.19 | 3.75 ± 0.21 | −5.5 |

For certified elements, reference values are certified value ± uncertainty. Measured values are mean ± SD.
Relative difference = 100 × (measured value − reference value) / reference value.

**Table S4. Mean RSD values (%) for triplicate instrumental measurements of cow-hair digests by F-AAS.**

| Parameter | Mn | Fe | Ni | Cu | Zn | Cd | Pb |
|---|---|---|---|---|---|---|---|
| RSD | 0.16 | 0.32 | 1.08 | 0.39 | 0.12 | 2.01 | 1.34 |

**Table S5. Comparison of certified and measured Hg mass fractions in BCR-414 plankton (mg/kg dw; n = 5).**

| Analyte | Certified value<br>Uncertainty | AMA-254 mean<br>SD | Relative difference (%) |
|---|---|---|---|
| Hg | 0.276<br>0.018 | 0.261<br>0.015 | −5.4 |

Values in mg/kg dw are numerically equivalent to the source unit µg/g dw.

SD, standard deviation.

Relative difference = 100 × (measured value − certified value) / certified value.

61. Thermo Fisher Scientific. 2011. iCE 3000 Series AA Spectrometers: Operator's Manual. Cambridge, UK. Available at: https://photos.labwrench.com/equipmentManuals/9291-6306.pdf (accessed 6 January 2024).
